# Huntingtin Interacts with the Cue Domain of gp78 and Inhibits gp78 Binding to Ubiquitin and p97/VCP

**DOI:** 10.1371/journal.pone.0008905

**Published:** 2010-01-26

**Authors:** Hui Yang, Chao Liu, Yongwang Zhong, Shouqing Luo, Mervyn J. Monteiro, Shengyun Fang

**Affiliations:** 1 Center for Biomedical Engineering and Technology, University of Maryland, Baltimore, Maryland, United States of America; 2 Department of Medical Genetics, Cambridge Institute for Medical Research, Cambridge, United Kingdom; Johns Hopkins School of Medicine, United States of America

## Abstract

Huntington's disease (HD) is caused by polyglutamine expansion in huntingtin (htt) protein, but the exact mechanism of HD pathogenesis remains uncertain. Recent evidence suggests that htt proteins with expanded polyglutamine tracts induce endoplasmic reticulum (ER) stress, probably by interfering with ER-associated degradation (ERAD). Here we report that mutant htt interacts and interferes with the function of gp78, an ER membrane-anchored ubiquitin ligase (E3) involved in ERAD. Mapping studies showed that the HEAT repeats 2&3 of htt interact with the cue domain of gp78. The interaction competitively reduces polyubiquitinated protein binding to gp78 and also sterically blocks gp78 interaction of p97/VCP, a molecular chaperone that is essential for ERAD. These effects of htt negatively regulate the function of gp78 in ERAD and are aggravated by polyglutamine expansion. Paradoxically, gp78 is still able to ubiquitinate and facilitate degradation of htt proteins with expanded polyglutamine. The impairment of ERAD by mutant htt proteins is associated with induction of ER stress. Our studies provide a novel molecular mechanism that supports the involvement of ER stress in HD pathogenesis.

## Introduction

Huntington's disease (HD) is an inherited neurodegenerative disorder that is caused by abnormal expansion of tri-nucleotide CAG repeats in the first exon of the gene encoding huntingtin (htt) protein [1]–[4]. The expanded CAG repeats are translated into different lengths of polyglutamine tracts: expansions greater than 35 repeats induce HD disease [4], [5], with the age of onset inversely related to the length of the polyglutamine expansion [3], [4].

Wild type htt protein is mostly localized in the cytoplasm with a small proportion found in the nucleus [6]. Subcellular fractionation studies have indicated that the protein is present in the endoplasmic reticulum (ER), Golgi, plasma membrane, endocytic and autophagic vesicles, endosomes, and mitochondria [6]–[13]. The function of htt protein is still unresolved. However, many functions have been attributed to the protein including a role in axonal transport, endocytosis, mitochondiral and vesicular trafficking, and gene transcription [12], [14]–[18]. The multitude of its possible functions raises the possibility that polyglutamine expansion might induce disease by interference of one or more of these functions [4]. However, substantial evidence suggests that the N-terminal htt fragments, harbouring the expanded polyglutamine tract, are a chief trigger of toxicity, largely through a gain-of-function mechanism [4], [19]. Several proteases, including certain caspases, calpain, and an unknown aspartic protease, cleave mutant htt within the region spanning amino acids 513–552, suggesting that these enzymes could play a role in disease pathogenesis [4], [20], [21]. While definitive evidence for how polyglutamine expansions in htt induce disease remains to be established, a range of different mechanisms have been implicated, including oxidative stress, transcriptional dysregulation, mitochondrial dysfunction, inhibition of the ubiquitin proteasome system (UPS), excitotoxicity, and impairments of axonal transport and synaptic transmission [4], [22], [23].

Recently, another mechanism that has gained attention is the finding that mutant htt induce ER stress by interference with ER-associated degradation (ERAD), the process by which misfolded proteins in the ER are exported to the cytosol for degradation by the proteasomes [24]–[29]. Complexes containing multiple proteins are required for efficient ERAD. ER membrane-anchored ubiquitin ligases (E3), an indispensible component of the ERAD complexes, ubiquitinates misfolded ER proteins, thus marking the proteins for elimination by proteasomes [30]. gp78 and Hrd1 are two well-characterized ER-localized E3s involved in ERAD in mammalian cells [31]–[38]. The cytosolic domains of gp78 and Hrd1 contain a RING finger domain that harbors the E3 activity [31], [39], as well as domains that recruit the AAA ATPase protein, p97/VCP, to the ERAD complex [32]–[34]. p97/VCP is an essentail factor that facilitates the dislocation of polyubiquitinated ER proteins to the cytosol for degradation [40], [41]. The recent reports showed that mutant htt interacts with p97/VCP and its cofactors Ufd1-Npl4 heterodimer and entraps them in mutant htt aggregates, which impairs ERAD [26].

Impairment of the ERAD machinery has also been reported in other neurodegenerative diseases. For example, mutant SOD1 (Superoxide dismutase 1), a protein mutated in familial Amyotrophic Lateral Sclerosis (ALS) disease, was shown to abnormally interact with the Derlin1 protein, an essential ERAD component believed to from the channel through which misfolded proteins are exported from the ER [42]–[44]. Mutant SOD1-Derlin1 interaction results in inhibition of ERAD and causes ER stress-induced neuronal degeneration [44]. ER stress-induced neuronal cell death has been implicated in many other neurodegenerative diseases, such as Parkinson's disease, Alzheimer's disease, prion disease, and other polyglutamine diseases [26], [44]–[46]. ER stress activates the unfolded protein response (UPR), which triggers apoptosis by up-regulation of CHOP/Gadd153 and activation of caspase-12, caspase-9, and caspase-3 [47], [48]. In addition, ER stress-induced neuronal death via activation of the Ire1-TRAF2-Ask1-JNK pathway has bee reported in models of ALS and in response to polyglutamine toxicity [49], [50].

We previously reported that Hrd1 ubiquitinates and targets mutant htt for degradation [51]. In this study, we identified gp78 as another novel interacting partner of htt. We demonstrated an interplay between mutant htt and gp78. Mutant htt compromises gp78's function in ERAD and triggers ER stress by directly binding to the cue domain, which results in inhibition of gp78 interaction with polyubiquitinated proteins and p97/VCP. On the other hand, gp78 ubiquitinats and enhances degradation of htt proteins with expanded polyglutamine repeats, but does not affect the degradation of htt proteins with normal polyglutamine repeats. These results support the role of ER stress in pathogenesis of HD and suggest that gp78 may play a protective role against mutant htt toxicity.

## Results

### Overexpression of Mutant htt Stabilizes gp78 Protein, Inhibits ERAD, and Induces ER Stress

**Figure 1:**
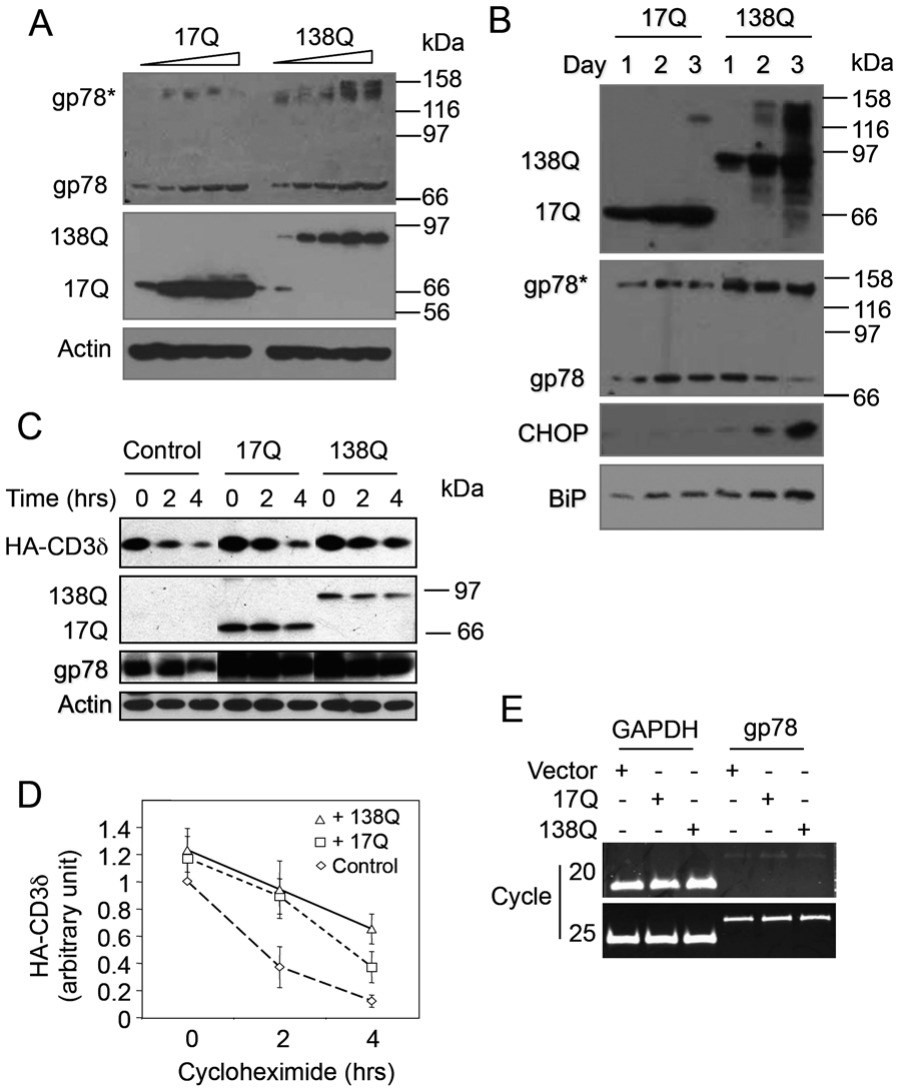
Nhtt overexpression upregulates gp78 protein and induces gp78 aggregation. A. Nhtt upregulates gp78 protein in a dose-dependent manner. Increasing amounts of Nhtt17Q and Nhtt138Q were expressed in HEK293 cells. Endogenous gp78 was examined by immunoblotting. Actin was blotted as a loading control. gp78* indicates aggregated gp78. B. Accumulation of Nhtt138Q causes increased aggregation of gp78 and induces ER stress. Equal amount of Nhtt17Q and Nhtt138Q were expressed in HEK293 cells. gp78, CHOP, and BiP were assessed in these cells by immunoblotting. C, D. Nhtt inhibits CD3δ degradation and polyglutamine expansion aggravates the inhibition. Nhtt17Q and Nhtt138Q were transfected into HEK293 cells that stably express CD3δ. CD3δ degradation in transfected cells was analyzed by cycloheximide (CHX) chase. D. Quantitative results of CD3δ degradation are expressed as mean +/− SD, *n* = 3. After 2 hrs: vector vs. 17Q or 138Q, *p*<0.05; after 4 hrs: vector vs. 17Q or 138Q, *p*<0.05; 17Q vs. 138Q, *p*<0.05, *t*-test. E. Neither Nhtt17Q nor Nhtt138Q affect gp78 mRNA expression. HEK293 cells expressing Nhtt17Q or Nhtt138Q were examined by RT-PCR of gp78 mRNA. GAPDH mRNA was amplified as a control.

Because mutant htt proteins have been found to induce ER stress [26], [27], [29], we examined if gp78 that is involved in ERAD might regulate htt-mediated toxicity. To test this possibility, we examined whether overexpression of htt proteins containing different number of polyglutamine repeats affects gp78 expression. Increasing amounts of cDNA encoding the N-terminal 588 amino acids fragment of htt containing either normal number (Nhtt17Q) or pathogenic number of polyglutamine (Nhtt138Q) were transfected into HEK293 cells. Sixteen hours later, the expression levels of gp78 were monitored by immunoblotting. We found that overexpression of both Nhtt17Q and Nhtt138Q led to a dose-dependent increase of gp78 protein. The effects caused by Nhtt138Q appeared to be more prominent, especially the slower migrating forms of the protein (indicated as gp78* in Fig. 1A). Previous studies have reported that the slower migrating forms represent aggregated gp78 protein [31]. In addition, a time-course study revealed a progressive accumulation of Nhtt17Q and Nhtt138Q proteins over time (Fig. 1B). Interestingly, accumulation of Nhtt138Q markedly increased accumulation of the aggregated form of gp78, which was accompanied by a time-dependent decrease of monomeric gp78 and an increase in induction of ER stress as monitored by CHOP and BiP expression (Fig. 1B). The correlation of gp78 aggregation with induction of ER stress suggests that Nhtt138Q expression may compromise ERAD. In support of this notion, cycloheximide chase experiments showed that expression of either Nhtt-17Q or Nhtt138Q inhibits the degradation of CD3δ (Fig. 1C, D), a known substrate of gp78, but Nhtt138Q inhibited more than that by Nhtt17Q. To exclude the possibility that mutant htt regulates gp78 expression at the transcriptional level, we examined expression of gp78 mRNA by RT-PCR analysis and found that overexpression of neither Nhtt17Q nor Nhtt138Q affected gp78 mRNA expression (Fig. 1E). These results suggest that overexpression of mutant htt compromises gp78 function in ERAD by causing accumulation of the non-functional form of gp78.

### gp78 Interacts with htt

**Figure 2:**
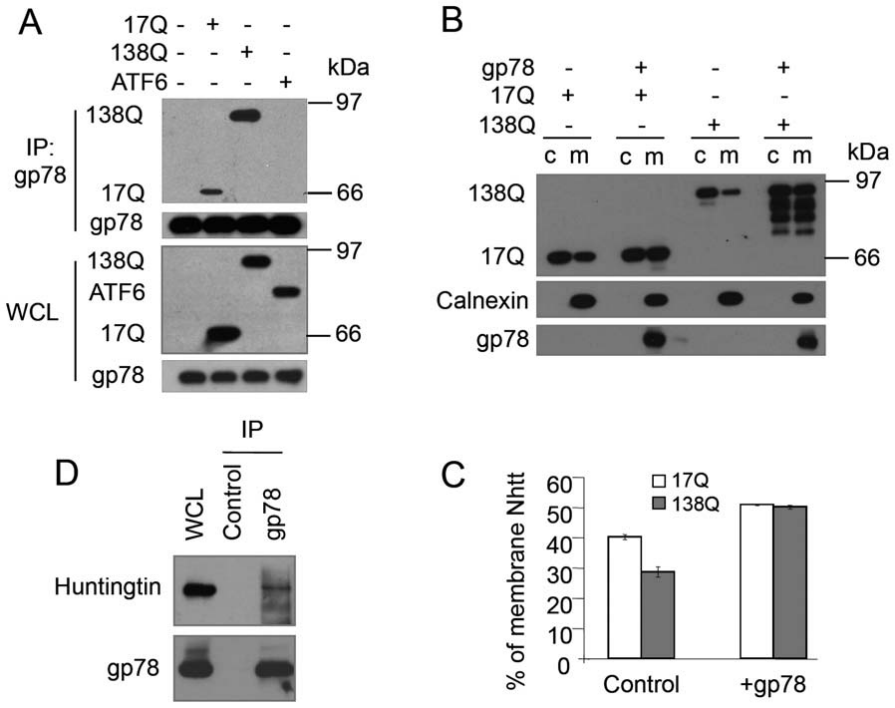
gp78 interacts with htt. A. gp78 interacts with Nhtt and polyglutamine expansion increases the interaction. Nhtt17Q and Nhtt138Q were expressed in HEK293 cells. FLAG-ATF6 and empty vector was used as controls. Immunoprecipitation was performed with anti-gp78 antibody followed by immunoblotting with the antibodies as indicated. B, C. gp78 enhances Nhtt association with the microsomes. HEK293 cells fractionated as indicated were subjected to fractionation into microsomal membranes (m) and cytosol (c). gp78 and Nhtt were detected by immunoblotting using anti-gp78 and anti-FLAG antibodies. Calnexin was blotted as an ER marker. Microsome-associated Nhtt was expressed as a percentage of total Nhtt associated with microsomes and in the cytosol (Mean ± SD, *n* = 3). D. Endogenous gp78 associates with htt. HEK293 cells were lysed in RIPA buffer and gp78 was immunoprecipitated with anti-gp78 antibody clone 2G5. Endogenous htt was detected by immunoblotting.

We reasoned that the differential modulation of gp78 accumulation by htt proteins might arise from the differences in interaction of the proteins. To test this possibility, FLAG-tagged Nhtt17Q, Nhtt138Q, or ATF6 that was as a negative control, were expressed in HEK293 cells and interaction of the proteins was examined after immunoprecipitation of gp78. Immunoblotting analysis revealed that Nhtt17Q and Nhtt138Q, but not ATF6 were co-immunoprecipitated with gp78 (Fig. 2A). Interestingly, gp78 associated with more Nhtt138Q than Nhtt17Q, suggesting that htt proteins with longer polyglutamine repeats bind more strongly with gp78 than those with shorter polyglutamine repeats (Fig. 2A). The increased association of Nhtt138Q with gp78 may account for the increased aggregation of gp78 (Fig. 1A, B) leading to more inhibition of CD3δ degradation by Nhtt138Q than Nhtt17Q as shown in Figure 1C and D. Because gp78 is an ER resident protein, we next examined if overexpression of gp78 affects htt binding to the ER. Thus, Nhtt17Q and Nhtt138Q were expressed individually, or co-expressed with gp78 in HEK293 cells. Transfected cells were then fractionated into cytosol and ER-containing microsomal membrane fractions, and htt protein partitioning in the two fractions was examined by immunoblotting. The results showed that overexpression of gp78 increased the amount of Nhtt17Q and Nhtt138Q that was present in microsomes (Fig. 2B, C), indicating that gp78 recruits htt to the ER membrane.

The results described above demonstrated that gp78 interacts with overexpressed htt proteins. To establish that the interaction is not an artifact of the overexpression of htt proteins, we examined endogenous protein interaction in HEK293 cells. Indeed, we found that endogenous htt was coprecipitated with gp78 (Fig. 2D). Taking together, the results suggest that gp78 normally interacts with htt proteins and that polyglutamine expansion enhances the interaction of the proteins.

### Htt, gp78, and p97/VCP Form a Trimeric Complex That Is Disrupted by Polyglutamine Expansion

**Figure 3:**
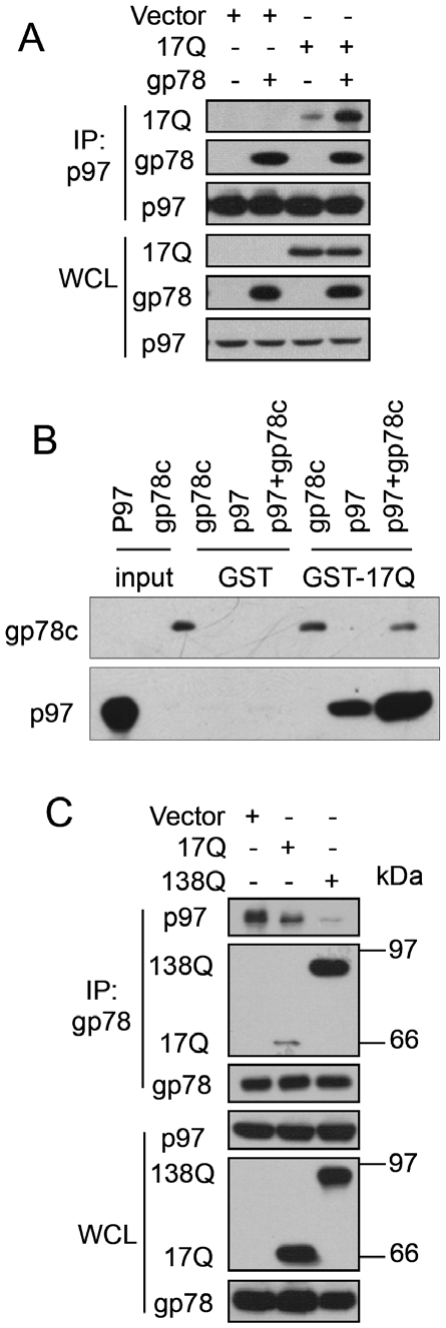
gp78 forms a complex with Nhtt and p97/VCP and Nhtt138Q disrupts gp78-p97/VCP interaction. A. gp78 enhances Nhtt17Q interaction with p97/VCP. HEK293 cells transfected as indicated were processed for IP for p97/VCP. Immunoprecipitates were blotted for the indicated proteins. B. Formation of trimeric complex of GST-Nhtt17Q, gp78c, and p97/VCP. GST-Nhtt17Q immobilized on beads was incubated with purified 6-His-p97/VCP and 6-His-gp78c. After washing, GST-Nhtt17Q-associated gp78c and p97/VCP were detected by immunoblotting. C. Nhtt inhibits gp78 interaction with p97/VCP and polyglutamine expansion aggravates the inhibition. HEK293 cells transfected as indicated were processed for immunoprecipitation for gp78. Precipitates were blotted for the indicated proteins.

To explore how mutant htt proteins might inhibit ERAD, we conducted additional immunoprecipitation experiments this time focusing on p97/VCP, a known gp78-binding protein [32], [52], which plays a critical role in coupling ubiquitination, dislocation, and proteasomal degradation of substrates during ERAD [53]–[55]. In these experiments, HEK293 cells were transfected with either the Nhtt17Q expression construct or its empty vector, alone, or cotransfected with gp78 cDNA after which p97/VCP was immunoprecipitated and examined for coprecipitation of the expressed proteins by immunoblotting. As shown in Figure 3A, a small amount of Nhtt17Q was coimmunoprecipitated with p97/VCP, with significantly more coimmunoprecipitation upon overexpression of gp78, suggesting that gp78 is important for mediating interaction of htt with p97/VCP (Fig. 3A). To determine whether htt interacts directly with gp78 and p97/VCP proteins, GST-pull down assays were performed using purified recombinant proteins. In one set of incubations, GST-Nhtt17Q was mixed with either purified 6-His-gp78 cytosolic tail (6-His-gp78c) or 6-His-p97/VCP alone or 6-His-gp78c and 6-His-p97/VCP proteins for 2 h at 4°C. A duplicate set of incubations was made with GST instead of GST-Nhtt17Q as a negative control. As shown in Figure 3B, 6-His-gp78c and 6-His-p97/VCP proteins bound to GST-Nhtt17Q, but not to GST. Interestingly, more 6-His-p97/VCP was pulled down by GST-Nhtt17Q from the mixture to which 6-His-gp78c was added (Fig. 3B), similar to the effect seen by coimmunoprecipitation in cells (Fig. 3A). These results suggest that htt, gp78, and p97/VCP form a trmieric complex through direct interactions.

We next investigated whether htt proteins containing different lengths of polyglutamine tracts affects binding of p97/VCP with gp78. For these assays HEK293 cells were transfected with either Nhtt17Q or Nhtt138Q expression constructs, or their empty vector, followed by immunoprecipitation of gp78 from the cells and immunoblotting for p97/VCP. The results revealed a polyglutamine length-dependent reduction of coimmunoprecipitation of p97/VCP with gp78 (Fig. 3C). These results suggest that htt may regulate gp78 and p97/VCP interaction under physiological conditions and that polyglutamine expansions might inhibit gp78 and p97/VCP interaction.

### A Region in HEAT 2 and HEAT 3 Repeats of htt Interacts with the Cue Domain of gp78

**Figure 4:**
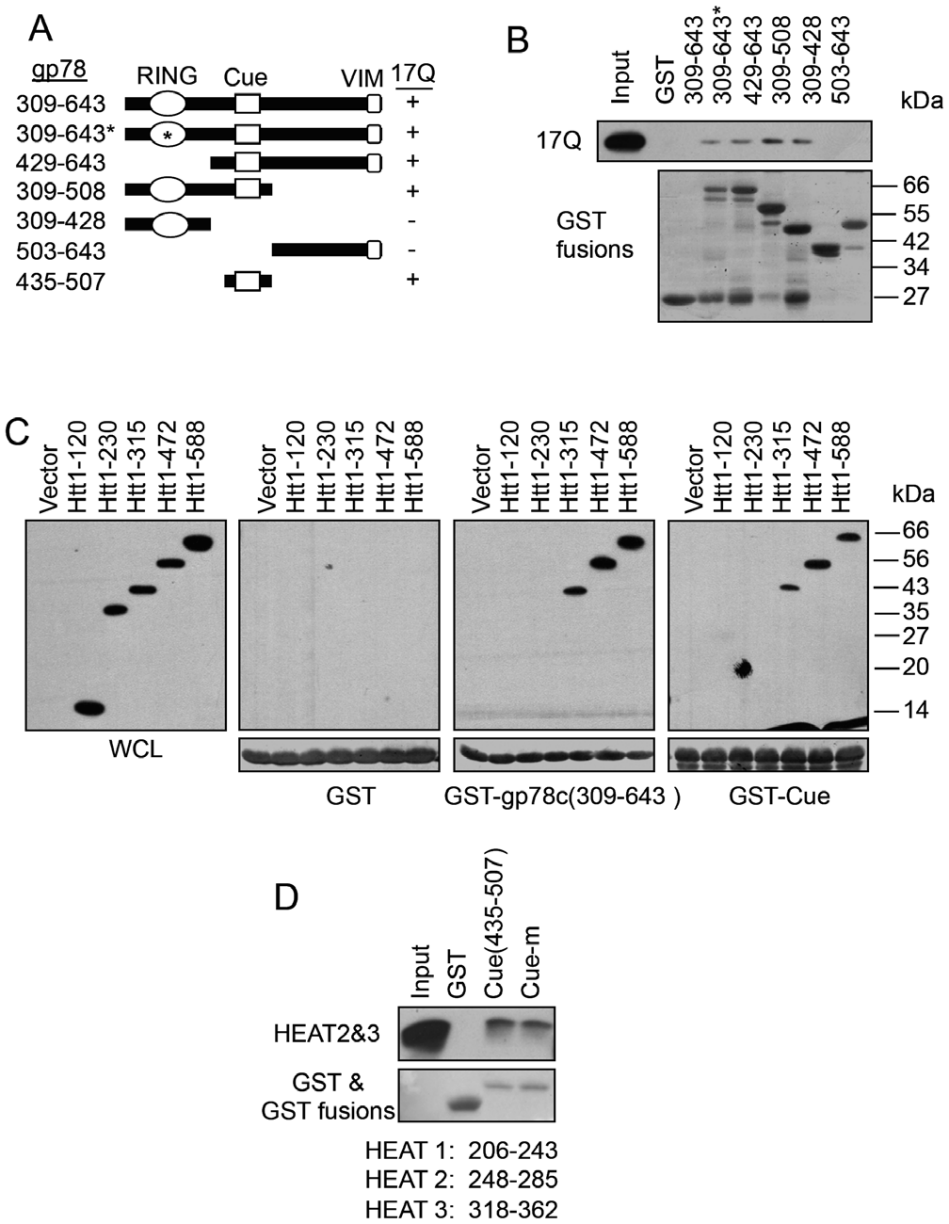
The cue domain of gp78 interacts with the HEAT 2 & 3 repeats of htt. A. The cue domain of gp78 is responsible for its interaction with htt. Left: Diagram shows GST-gp78c and its truncation mutants used in pull down assay. Right: GST-gp78c and its truncation mutants were incubated with HEK293 cell lysates expressing Nhtt17Q. The Nhtt17Q associated with GST-gp78 was detected with anti-FLAG antibody. GST fusion proteins were stained by ponceau s red. B. The cue domain of gp78 interacts with a region containing the HEAT 2&3 repeats of htt. HEK293 cell lysates expressing various Nhtt truncation mutants containing 17Q were subjected to GST pull down experiment with GST-gp78c and its mutants. Nhtt mutants were detected by immunoblotting. C. Direct interaction of the cue domain of gp78 and the HEAT 2&3 repeats. Recombinant 6His-tagged HEAT 2&3 protein was incubated with GST, GST-cue domain or a GST-Cue domain mutant (Cue-m, see Figure 5A for detailed amino acid changes). Binding of HEAT 2&3 was detected by immunoblotting for 6-His. GST and its fusion proteins were stained by ponceau s red.

To gain more insight into the molecular mechanism of interaction between htt and gp78, we used GST pull-down assays to map the binding site in each protein. The binding site of htt in gp78 was mapped by incubating cell lysates prepared from Nhtt17Q-expressing cells with GST-fusion proteins containing different portions as well as mutated variants of gp78. As shown in Figure 4A, Nhtt17Q binding was mapped to a region containing the cue domain of gp78. We next determined the site of gp78 binding in htt protein. To do so, we transfected a series of constructs encoding progressive C-terminal truncations of a FLAG-tagged N-terminal 1-588 amino acid fragment of htt into HEK293 cells and used lysates from the cells to examine if the FLAG-tagged proteins bind GST, GST-gp78c, or GST-gp78-cue domain fusion proteins by pull-down assays. As shown in Figure 4B, the minimal N-terminal htt fragment that bound GST-gp78c and GST-gp78-cue domain fusion proteins was the amino acid 1-315 of htt, although binding of this fragment was noticeably less than that mediated by the larger 1-472 and 1-588 fragments. By contrast to these fragments, no binding was observed with htt fragments of amino acid 1-230, or smaller. An examination of the amino acid sequence of the htt fragment involved in gp78 binding revealed that it contains three HEAT repeats (aa 206 to 243, 248 to 285, and 318 to 362), which we considered as candidates for mediating gp78 binding. HEAT repeats are thought to form α-helical bundles that appear to function in protein-protein interaction [4]. We concentrated on HEAT repeats 2 and 3, but not repeat 1, because the former, but not latter were contained in htt fragments involved in gp78 binding. Accordingly, we analyzed whether purified 6-His-htt-aa 245–370 fusion protein that contains HEAT repeat 2 and 3 bind GST-gp78-cue domain or GST. The results showed that 6-His-htt-aa 245–370 binds to GST-gp78-cue domain and its mutant (cue-m: two mutated ubiquitin-interacting residues - M467T and L477D), but not GST (Fig. 4C). Collectively, these data suggest that gp78 and htt bind directly through interactions between the cue domain of gp78 and the HEAT 2 and 3 repeats of htt.

### HttN Inhibits Polyubiquitin Binding to gp78

**Figure 5:**
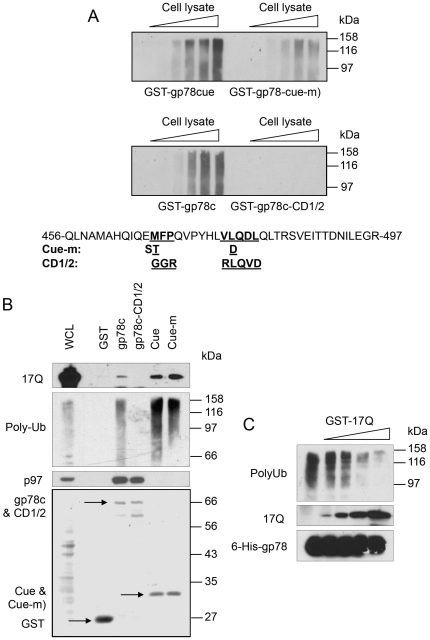
Htt588 inhibits polyubiquitinated protein binding to gp78. A. Effects of cue domain mutation on polyubiquitin binding. GST fusions of two different cue domain mutants: cue-m and gp78cCD1/2(aa309-643), and their corresponding wt controls, cue and gp78c(aa309–643), were immobilized on beads and incubated with increasing amounts of lysates prepared from HEK293 cells. After washing, polyubiquitinated proteins associated with these GST fusion proteins were examined by immunoblotting for ubiquitin. B. gp78c-CD1/2 fails to bind both Nhtt17Q and polyubiquitin. GST fusion proteins as indicated were incubated with HEK293 cell lysates prepared from HEK293 cells transfected with Nhtt17Q. Polyubiuitinated proteins and Nhtt17Q were detected by immunoblotting. C. Nhtt17Q dose-dependent inhibition of polyubiquitinated protein binding to 6-His-gp78c. Beads-immobilized 6-His-gp78c was incubated with HEK293 cell lysates or along with increasing amounts of purified GST-Nhtt17Q. After washing, beads-associated polyubiquitin and GST-Nhtt17Q were determined by immunoblotting.

Previous reports have indicated the cue domain of gp78, which is known to bind ubiquitin, is required for gp78 function in ERAD [32], [56], [57]. Based on structural and bioinformatics information of cue domain [58], [59], we mutated three residues (EML) that appeared to be involved in ubiquitin binding to residues (STD, see Fig. 5A) that we considered would diminish, but may not eliminate, ubiquitin binding (Fig. 5A, we called the triple mutant Cue-m). To experimentally validate the effects of these mutations, we compared ubiquitin binding between GST proteins containing the wild-type gp78-cue domain with that of the cue-m in pull-down assays. We also analyzed a more severely crippled cue mutant of gp78 (mutated in six different positions, called CD1/2 [56], see Fig 5A) that is devoid of ubiquitin binding. As shown in Figure 5A, polyubiquitin binding to the cue-m was significantly reduced, but was not abolished, compared to the binding seen with the wild type and CD1/2 mutant proteins (Fig. 5A, upper panel).

Having established the polyubiquitin binding properties of the three gp78-containing cue proteins, we next tested them for their ability to bind Nhtt17Q protein by pull-down assays. These assays revealed that the binding between Nhtt17Q and gp78 was retained by the proteins containing either the intact or cue-m version of the cue domain, but completely abolished in the cue-CD1/2 mutant that is also unable to bind polyubiquitin chains (Fig. 5B). These results suggest that the HEAT2 and 3 repeats may bind to the same residues in the cue domain that are required for ubiquitin interaction. To obtain evidence in support of this idea, we asked whether htt inhibits ubiquitin-binding to the cue domain in a competition experiment. In this experiment, His-tagged gp78c was immobilized on Ni-NTA beads and incubated with a constant amount of HEK293 cell lysate together with increasing amounts of purified GST-Nhtt17Q. The beads were then washed and binding of GST-Nhtt17Q and polyubiquitin was monitored by immunoblotting. As shown in Figure 5C, the amounts of polyubiquitin pulled down by gp78c decreased upon addition of GST-Nhtt17Q, in a dose-dependent manner. This data indicates that htt competes with ubiquitin binding to gp78.

**Figure 6:**
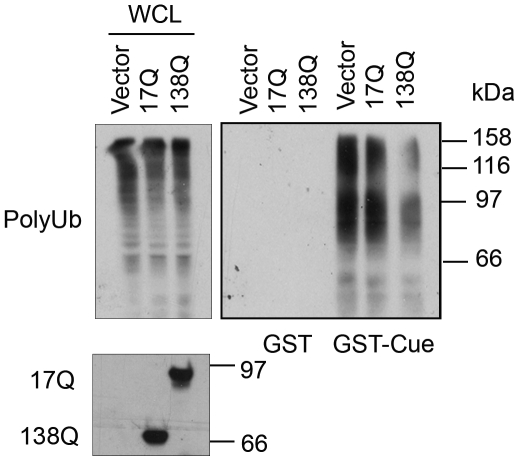
Effects of Nhtt17Q and Nhtt138Q on polyubiquitinated protein binding to the cue domain of gp78. Nhtt138Q inhibits polyubiquitin binding to gp78 more than Nhtt17Q. Lysates prepared from HEK293 cells expressing equal amount of Nhtt138Q and Nhtt17Q were used in GST-cue pull down assay. Association of polyubiquitinated proteins with GST or GST-cue was determined by anti-ubiquitin blotting.

Because gp78 appeared to bind more Nhtt138Q protein than with the Nhtt17Q protein (Fig. 2A, 3C), we suspected that Nhtt138Q would hinder polyubiquitin interaction with gp78 more efficiently compared with Nhtt17Q. To test this possibility, lysates prepared from HEK293 cells expressing equal amount of Nhtt17Q and Nhtt138Q were incubated with either GST-cue protein or GST alone. The GST proteins were then affinity-purified and the amount of polyubiquitin proteins that was associated with the complex was analyzed by immunoblotting. Consistent with our prediction, polyubiquitin binding to GST-cue was diminished in a polyglutamine dependent manner (Fig. 6). As expected, polyubiquitin did not bind to the GST alone. Functionally, Nhtt138 also inhibits CD3δ degradation more efficiently than Nhtt17Q (Fig. 1C, D). These results are consistent with the previous reports that polyubiquitin binding to the cue domain of gp78 is essential for gp78's function in ERAD [56], [57]. Thus, the reduced binding of polyubiquitinated proteins to the cue domain in httN138Q-containing lysates may be resulted from the increased binding of httN to the cue domain due to polyglutamine expansion.

### Anti-Cue Domain Antibody Is Sufficient to Inhibit gp78 Binding to Polyubiquitin and p97/VCP

**Figure 7:**
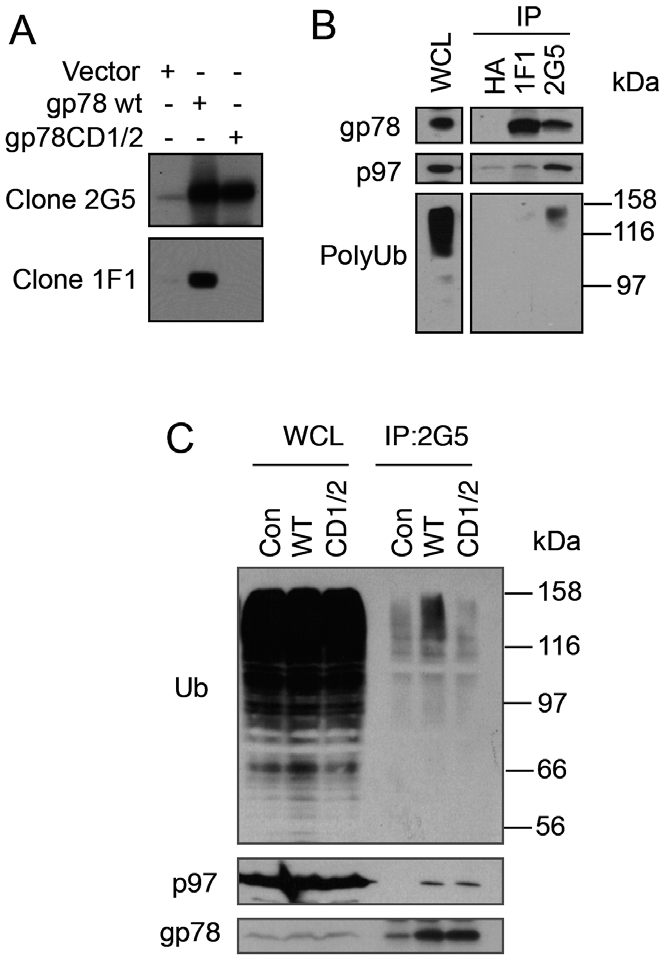
Anti-cue domain antibody fails to co-immunoprecipitate p97/VCP and polyubiquitinated proteins. A. Monoclonal anti-gp78 antibody clone 1F1 does not recognize gp78CD1/2. HEK293 cells expressing wt gp78 and gp78CD1/2 were subjected to immunoblotting with anti-gp78 antibody clone 2G5 and 1F1, respectively. B. 1F1 precipitates gp78 but not p97/VCP and polyubiquitinated proteins. gp78 was immunoprecipitated from HEK293 cell lysates with 2G5 and 1F1, respectively. Co-immunoprecipitation of p97/VCP and polyubiquitinated proteins was examined by immunoblotting. C. gp78CD1/2 interacts with p97/VCP but not polyubiquitinated proteins. HEK293 cells transfected with wt gp78 or gp78CD1/2 was processed for immunoprecipitation with 2G5 antibody.

The preceding results suggested that interaction of htt with the cue domain of gp78 leads to an obstruction in the binding of polyubiquitin and p97/VCP to gp78, which is required for gp78 function in ERAD. To gain more insight into the mechanism of the inhibition, we evaluated whether physical masking of the cue domain with an antibody specific for the domain would likewise block polyubiquitin and p97/VCP binding to gp78. This approach became feasible because of the properties of monoclonal antibodies that we possessed. One of them, called 2G5 recognizes an epitope between amino acids 497 to 578 of gp78, which is not part of the cue domain. The antibody recognizes gp78, but does discriminate between the wild type and CD1/2 cue mutant proteins (Fig. 7A). A second monoclonal antibody called 1F1, specific against the cue domain of gp78 (Fig. S1), differed in that it fails to bind gp78CD1/2 protein (Fig. 7A). We utilized the two antibodies to immunoprecipitate gp78 from cells, together with an anti-HA antibody as a negative control, and probed the precipitates to see whether p97 and ubiquitinated proteins were coimmunoprecipitated. As shown in Figure 7B, gp78 was precipitated by both 2G5 and 1F1 antibodies, but not by the anti-HA antibody, as expected. Further blotting analysis of the gp78 precipitated materials revealed that p97/VCP and polyubiquitinated proteins were coimmunoprecipitated by the 2G5 antibody, but not by the 1F1 antibody (Fig. 7B). These results suggest that, 1) inhibition of polyubiquitin binding is sufficient to inhibit gp78-p97/VCP interaction, or 2) htt and anti-cue domain antibody binding sterically blocks p97/VCP binding to its binding site [the p97/VCP-Interacting Motif (VIM)] in gp78 [52]. To test these possibilities, we expressed gp78CD/1/2 and wt gp78, respectively, in HEK293 cells. Co-immunoprecipitation showed that gp78CD1/2 binds to p97/VCP as well as wt gp78 (Fig. 7C). Thus, polyubiquitin binding does not affect gp78-p97/VCP interaction, supporting the idea that htt binding to cue domain not only inhibits ubiquitin binding but also sterically blocks the access of VIM to p97/VCP.

### gp78 Promotes Nhtt138Q Degradation

**Figure 8:**
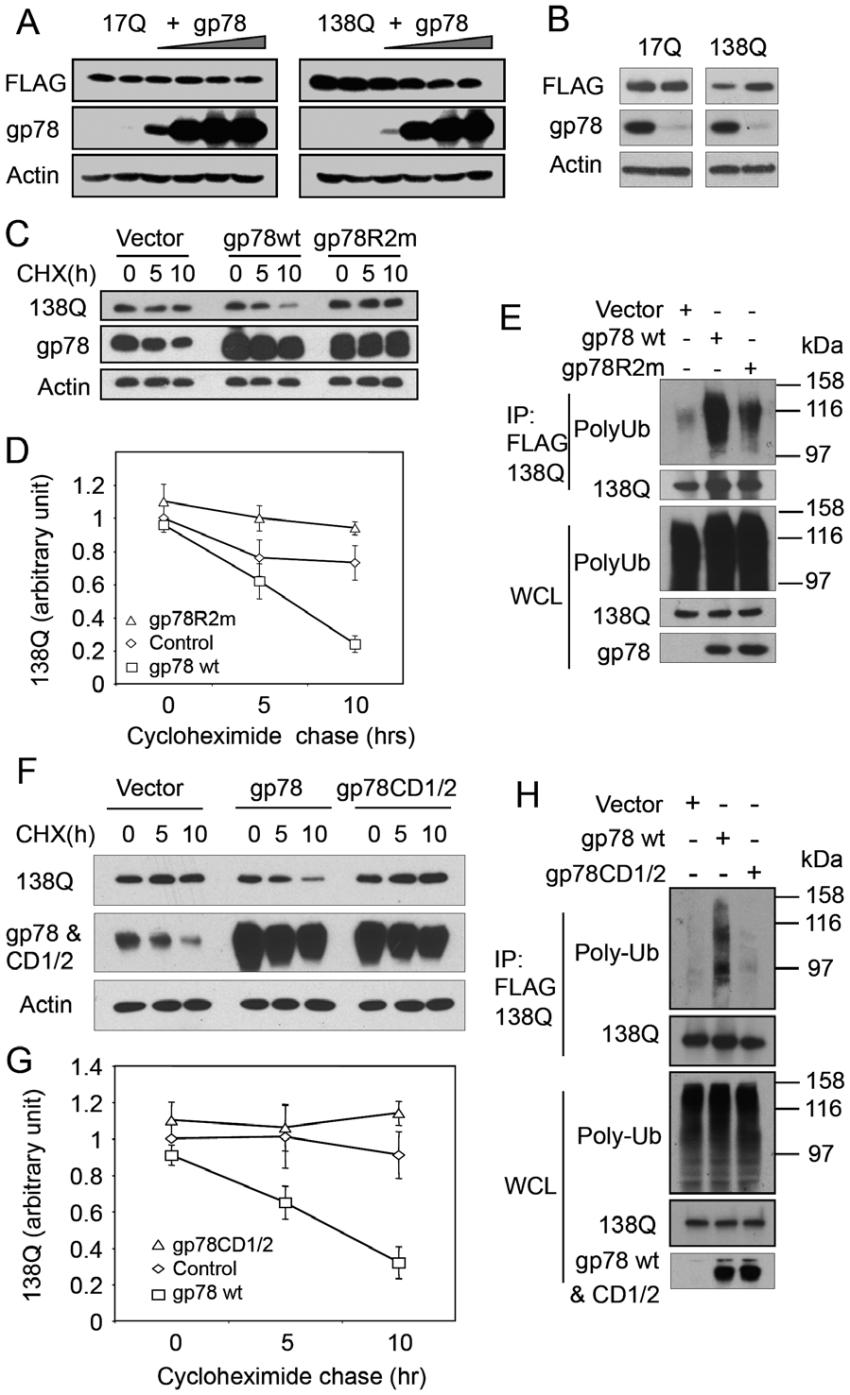
gp78 ubiquitinates and targets Nhtt138Q but not Nhtt17Q for degradation dependent on its RING finger and cue domain. A. Overexpression of gp78 decreases the levels of Nhtt138Q but not Nhtt17Q protein. Increasing amounts of gp78 were expressed in HEK293 cells that had been transfected with Nhtt17Q or Nhtt138Q. 24 h after gp78 transfection, cells were processed for immunoblotting for the indicated proteins. B. Knockdown of gp78 stabilizes Nhtt138Q but not Nhtt17Q. gp78 was knocked down by RNAi in HEK293 cells expressing Nhtt138Q or Nhtt17Q. 2 days after the knockdown cells were processed for immunoblotting for gp78 and Nhtt17Q and Nhtt138Q. Actin was blotted as a loading control. C. gp78 enhances Nhtt138Q degradation, which requires an intact RING finger. HEK293 cells transfected as indicated were subjected to cycloheximide (CHX) chase. Nhtt138Q degradation was quantified and expressed as mean +/− SD, *n* = 3, in D. E. gp78 ubiquitinates Nhtt138Q. Nhtt138Q-expressing HEK293 cells were transfected with wt gp78, or gp78R2m, or empty vector as a control. Nhtt138Q was immunoprecipitated with anti-FLAG antibody. Precipitates were processed for immunoblotting for ubiquitin. F - H. Cue domain is required for gp78 to ubiquitinate and target Nhtt138Q for degradation. Wt gp78, gp78CD1/2, or empty vector-transfected HEK293 cells were subjected to analysis as described in C–E.

We previously reported that the gp78-related E3 Hrd1 ubiquitinates and facilitates mutant htt degradation [51]. Physical interaction between gp78 and htt prompted us to investigate whether gp78 plays a similar role. First, we evaluated whether overexpression of gp78 affects the steady-state accumulation of htt. Nhtt17Q or Nhtt138Q was co-transfected with increasing amounts of gp78 into HEK293 cells. Figure 8A showed that gp78 overexpression induced a dose-dependent reduction in Nhtt138Q levels, but did not affect Nhtt17Q levels. To examine whether endogenous gp78 regulates htt levels, we used RNA interference to reduce gp78 expression. siRNA specific for gp78 was transfected into HEK293 cells that express Nhtt17Q or Nhtt138Q. siRNA that does not target any known gene was transfected as a negative control. Immunoblotting revealed that gp78 was efficiently knocked down by the appropriate siRNA, and this correlated with a noticeable increase in Nhtt138Q accumulation and almost no change in Nhtt17Q accumulation, as compared with the control siRNA-transfected cells (Fig. 8B). These data suggest that gp78 preferentially facilitates mutant htt degradation.

We next studied the consequence of overexpression of wild type and an E3 inactive mutant of gp78 on the turnover of Nhtt138Q protein by cycloheximide chase experiments. Thus, Nhtt138Q was co-expressed in HEK293 cells with vector control, wt gp78, or gp78R2m, an E3-inactive mutant gp78 [31]. 24 hour after transfection, cycloheximide was added to the cultures to inhibit protein synthesis and the turnover of Nhtt138Q was analyzed by immunoblotting of the cell lysates collected at 0, 5, and 10 h. As is evident from Figure 8C & D, overexpression of wt gp78 but not gp78R2m enhanced Nhtt138Q degradation, indicating that gp78 E3 activity is critical for promoting Nhtt138Q degradation. We then examined whether gp78 enhances Nhtt138Q ubiquitination. HEK293 cells were transfected as in Figure 8C and Nhtt138Q was immunoprecipitated from SDS-denatured cell lysates. Precipitates were processed for immunoblotting with anti-ubiquitin antibody. As expected, co-expression with wt gp78 but not gp78R2m increased polyubiquitination of Nhtt138Q. In contrast, overexpression of wt gp78 or gp78CD1/2 had no effects on either ubiquitination or degradation of Nhtt17Q (Fig. S2). These results suggest that gp78 acts as an E3 and selectively targets mutant htt for degradation.

Because the cue domain of gp78 is instrumental for interaction with htt, we next examined whether an intact cue domain is required for Nhtt138Q degradation. Accordingly, Nhtt138Q was co-transfected with vector, wt gp78, or the gp78CD1/2 cue domain mutant that does not bind to Nhtt138Q and the turnover of Nhtt138 compared by cycloheximide chase experiments. As shown in Figure 8F-H, overexpression of gp78CD1/2 failed to promote Nhtt138Q ubiquitination and degradation compared to that seen with the wild type gp78 protein. Collectively, these observations suggest that gp78 functions to promote mutant htt degradation.

## Discussion

ER stress is triggered when the production of misfolded proteins in the ER exceeds the capacity of the organelle to refold or dispose the misfolded proteins [46], [60], [61]. The disposal of misfolded proteins from the ER is achieved by ERAD, the main pathway by which misfolded proteins are exported to the cytosol for degradation by proteasomes [62]. In this report we demonstrate that polyglutamine expansions in htt proteins induce ER stress by interfering with the function of gp78, an ER resident E3 that normally functions in ERAD. Our findings suggest that perturbation of gp78 function occurs by three interrelated mechanisms: 1) polyglutamine expansion enhances mutant htt interaction with the cue domain of gp78, thereby severely hindering polyubiquitin binding to the cue domain; 2) mutant htt sterically blocks p97/VCP interaction with gp78; 3) accumulation of mutant htt causes aggregation of gp78. In addition, we showed that gp78 is able to ubiquitinate and enhance mutant htt degradation. This function makes gp78 vulnerable when mutant htt accumulates and aggregates during HD progression.

The function of ERAD complexes is centered on ER membrane-spanning RING finger ubiquitin ligases, such as gp78, Hrd1, and RMA1 [31], [33], [34], [39], [63]. The RING finger of these E3s and the protein degradation machinery, proteasomes, are both localized on the cytosolic surface of the ER [30], [51], [62]. This spatial arrangement is thought to facilitate the coupling of machineries required for ubiquitination and retrotranslocation of ER substrates with proteasomal degradation. The arrangement also permits the complex to function in the disposal of misfolded proteins that normally reside in the cytosol. This dual role could have consequences for a cell because selection of a cytosolic substrate over a ER substrate by the ERAD complex could lead to a build up of misfolded proteins in the ER, especially when the cytosolic substrate is hard to be degraded.

The involvement of ER resident E3 in degradation of cytosolic substrates is illustrated by the interaction and degradation of mutant htt by Hrd1 [51]. Similarly, recent studies have shown that gp78 is able to enhance degradation of mutant SOD1 and ataxin-3 proteins [64]. Interestingly, mutant SOD1 also inhibits ERAD by interacting with Derlin1, a protein implicated as forming the channel through which misfolded proteins are dislocated from the ER during ERAD [42]-[44]. Our findings adds to this growing list, as we now demonstrate that gp78 promotes degradation of mutant htt. These data strongly support that ER-anchored E3 containing complexes play a quality control role not only for ER proteins, but also for non-ER proteins that are accessible from the cytosol as suggested previously [51]. As a response to accumulation of misfolded ER proteins, the expression of gp78 is increased by accumulation of neurodegenerative disease proteins, such as mutant htt, SOD1, and ataxin-3 [64]. This may represent a protective response to enhance the removal of these disease proteins. Indeed, gp78 not only facilitates the degradation of mutant htt, it also enhances the clearance of mutant SOD1 and ataxin-3 [64]. This is similar to the apparent broad specificity of ERAD E3s in targeting different proteins in the ER for degradation. On the other hand, a particular substrate can be degraded by multiple E3s. This is evident by the fact that mutant htt proteins can be degraded by either gp78 (this study) or Hrd1 [51]. We speculate that the apparent promiscuous substrate specificity of gp78 (and possibly other E3s) for the different neurodegenerative proteins would negatively impact ERAD because of the inefficiency in degradation of misfolded disease proteins. For example, mutant htt accumulates and gradually aggregates in neurons during HD progression, probably because the rate of mutant htt degradation is slower than the rate of its production/accumulation. The inefficiency in degradation of mutant htt proteins would preoccupy E3 proteins like gp78 and Hrd1 that might typically engage in ERAD in a futile effort toward degrading mutant htt proteins. We speculate that this non-productive interaction might lead to an accumulation of misfolded proteins in the ER leading to ER stress. This is supported by the fact that accumulation of mutant htt causes marked aggregation of gp78 protein while reduces monomeric gp78, which correlates with increased ER stress (Fig. 1B). gp78 aggregation is expected to diminish its function in ERAD.

In this study, we mapped the interaction between mutant htt and gp78 to the HEAT 2&3 repeats of htt and the cue domain of gp78. Htt with partial truncation of HEAT 3 still bound gp78 albeit to a lesser extent than a htt fragment with both HEAT 2 and 3 repeats. However, due to the insolubility of HEAT 2 fragment, we were not able to determine whether HEAT 2 is sufficient for binding to the cue domain. The region of htt that is involved in gp78 binding is consistent with our findings that N-terminal htt fragments bind gp78 irrespective of the number of polyglutamine repeats (i.e. both Nhtt17Q and Nhtt138Q bind gp78). The importance of this interaction is strengthened by our findings that endogenous htt was co-immunoprecipitated with endogenous gp78, suggesting it might serve a regulatory role for gp78. Future studies will be directed to study the functional importance of gp78 and htt interaction. Our results suggest that htt, gp78, and p97/VCP may form a ternary complex. However, p97/VCP forms hexamer and gp78 has also been shown to form a large oligomer [65]. Whether or not the ternary complex forms in cells may depend on the relative amounts of the proteins and may be subjected to regulation. This may partially explain why httN overexpression disrupted gp78-p97/VCP interaction in cells.

The cue domain of gp78 is well known for its ability to bind ubiquitin [32], [56], [57]. We found that htt and ubiquitin may share the same binding residues in the cue domain. This is supported by the fact that mutation of the residues in the cue domain that are known for ubiquitin interaction results in loss of htt binding. Furthermore, htt competes with polyubiquitin binding to the cue domain. In addition, htt binding appears to sterically block p97/VCP binding to gp78, which is supported by our studies showing an anti-cue domain antibody also appears to inhibit gp78 binding to polyubiquitin and p97/VCP. These results lead us to speculate that htt may be normally a negative regulator of gp78 function. However, this regulatory function is exaggerated by polyglutamine expansion because mutant htt enhances htt binding to gp78 leading to severer inhibition of gp78 interaction with polyubiquitin and p97/VCP, which may explain at least partially the inhibitory effects of mutant htt on ERAD. Paradoxically, despite interference of p97/VCP binding to gp78, mutant htt proteins are still degraded by gp78, albeit inefficiently. This might not be surprising because p97/VCP binding is principally thought to be required for providing the driving force for dislocation of substrates form the ER and might not be essential for gp78-mediated degradation of cytosolic substrates like mutant htt proteins. However, we cannot rule out the possibility that p97/VCP may act as a chaperone in the cytosol to deliver ubiquitinated mutant htt for degradation.

gp78 selectively targets Nhtt588-138Q but not Nhtt588-17Q for degradation, suggesting that polyglutamine expansion-induced misfolding renders htt a substrate for gp78. But recognition of misfolded htt by gp78 still requires the cue domain, since mutation of the cue domain that abrogates gp78-htt interaction failed to target mutant htt for degradation. Interestingly, the cue domain of gp78 has been previously reported to recognize another substrate, ΔF508CFTR [57], [66]. The finding that the HEAT 2&3 repeats provide a recognition site for gp78 might help explain why polyglutamine-expanded htt exon-I fragment that is widely used for studying mutant htt toxicity and lacks HEAT repeats 2&3, is not a substrate for gp78 as described in this study and reported recently by others [64]. However, our results do not distinguish whether mutant htt proteins lacking HEAT repeats 2&3 might interfere with normal gp78 function at different stage of HD. For example, recent studies reported that polyglutamine-expanded exon I entraps the p97/VCP-Ufd1-Npl4 complex in its aggregates and impairs ERAD [26]. Because the critical role of p97/VCP in gp78-mediated ERAD, the entrapment of p97/VCP in htt exon-I aggregates is expected to be detrimental to gp78 function. Taking together, our results provide novel insights into the role that gp78 might play in the turnover and pathogenicity of normal and mutant htt proteins.

## Materials and Methods

### Plasmids and Antibodies

cDNA encoding aa309-643 of gp78 was cloned into the pET-28a(+) vector to make pET28a(+)gp78c. cDNA encoding HEAT 2&3 (aa245–370) of human htt was cloned into pET28a(+) vector to generate pET28a(+)-HEAT 2&3 through BamHI and HindIII sites. cDNA encoding aa435-507 of mouse gp78 was cloned into pGEX4T1 vector to produce pGEX4T1-gp78-cue via BamHI and NotI sites. pGEX4T1-gp78-cue-m was generated by site-directed mutagenesis on pGEX4T1-gp8-cue. pCIneo-gp78CD1/2 is a generous gift from Dr. Allan M. Weissman [56]. The following are a list of plasmids that have been previously reported [32], [51], [67]: pQE9-p97/VCP, pCIneo-gp78, pCIneo-gp78R2m; pCI 3X Flag-Nhtt-588aa-17Q and pCI 3X Flag-Nhtt-588aa-138Q, pCI 3X Flag-Nhtt-120aa-17Q, pCI 3X Flag-Nhtt-230aa-17Q, pCI 3X Flag-Nhtt-315aa-17Q, pCI 3X Flag-Nhtt-472aa-17Q, pGEX4T-htt588aa; pGEX4T2-gp78C and its mutants.

Mouse monoclonal antibodies against HA, FLAG, and actin were purchased from Sigma. Mouse monoclonal anti-ubiquitin and anti-huntingtin antibodies were acquired from Santa Cruz Biotechnologies Inc. Monoclonal anti-p97/VCP was obtained from Affinity Bioreagents. Monoclonal anti-gp78 antibody clone 2G5 was reported previously [52] and clone 1F1 was recently characterized in the PI's lab as described in figure S1. pSuper-siRNA targeting gp78 was previously described [32].

### Cells Culture and Transfection

HEK293 cells were cultured in DMEM (Dulbecco's modified Eagle's medium) supplemented with 10%(V/V) fetal bovine serum, 50 units/ml penicillin, 50 ug/ml streptomycin and Glutamine under 5% CO2 in a humidified incubator. Transient transfections were performed using Lipofectamine 2000 (Invitrogen) or Calcium phosphate precipitation. HA-CD3 stable cell line has been described previously [52].

### Recombinant Proteins and GST-Pull-Down Assay

Bacterially expressed recombinant GST fusion proteins and 6-His-tagged proteins were produced as reported previously [32]. For mapping the huntingtin interaction site with gp78, we first immobilized 2 µg of each GST-gp78c(aa309–643) and its truncation mutants on glutathione-sepharose beads. Then we prepared lysates from cells transfected with FLAG-Nhtt17Q using lysis buffer containing 150 mM NaCl, 20 mM Tris/HCl, pH 7.5, 1 mM EDTA, and 0.5% NP-40. Beads-immobilized GST-gp78c and its mutants were incubated with FLAG-Nhtt17Q-containing lysates at 4°C for 2 h. After washing with the lysis buffer for 3 times, beads-bound Nhtt17Q was examined by immunoblotting. To map gp78 binding site on htt, HEK293 cells were transfected with plasmids encoding various truncation mutants of htt tagged with FLAG as described in Figure 4B. Lysates were prepared from these cells and incubated with beads-immobilized GST-gp78c or GST-cue domain at 4°C for 2 h. GST pull-down of polyubiquitinated proteins was performed similarly as described above. Direct interactions among gp78, htt, and p97/VCP were carried out using purified recombinant proteins and the binding procedure has been reported previously [52].

### Immunoblotting and Immunoprecipitation

Immunoblotting procedure used is essentially the same as we reported previously [52]. HEK293 cells were seeded at 6×10^5^ per well in 6-well plates prior to the day of transfection. Transfection was done with lipofectamine 2000 (Invitrogen) or by calcium phosphate precipitation. Twenty-four hours after transfection, cells were lysed in 2% SDS by boiling for 10 minutes. The resulted lysates were used for blotting of total proteins. To evaluate the effects of gp78 on degradation of Nhtt, 100 µg/ml cycloheximide (CHX) was added to the medium sixteen to twenty hours after transfection and the cells were subject to chase for 0, 5, and 10 hours before being processed for immunoblotting to detect the indicated proteins.

To determine protein-protein interactions by co-immunoprecipitation, HEK293 cells transfected as indicated in figures were lysed in buffer containing 150 mM NaCl, 20 mM Tris, pH 8.0, 5 mM EDTA, 0.5% NP40 and 1X protease inhibitor cocktail. The lysates were then clarified by centrifugation. The resulted supernatants were used for immunoprecipitation followed by immunoblotting as described in figure legends. For endogenous protein interaction, co-immunoprecipitation was done in HEK293 cell lysates without any transfection.

To assess if gp78 mediates ubiquitination of mutant htt in cells, HEK293 cells were co-transfected with plasmids encoding at gp78, gp78R2m, or gp78CD1/2 along with FLAG-Nhtt17Q or -Nhtt138Q. Twenty-four hours after transfection, cells were lysed in RIPA buffer (1X PBS, 1% Nonidet P-40, 0.5% sodium deoxycholate, and 10 mg ml phenylmethylsulfonyl fluoride) containing 1% SDS plus brief sonication. The lysates were clarified by centrifugation at 14,000 rpm at 4°C for 15 minutes and supernatants were diluted with 1X PBS to a final concentration of 0.2% SDS. FLAG-Nhtt was immunoprecipitated with anti-FLAG antibody using protein A-Sepharose beads. The ubiquitinated Nhtt was determined by immunoblotting for ubiquitin.

To assess the effects of gp78 on Nhtt-membrane association, HEK293 cells were co-transfected with gp78 and FLAG-Nhtt17Q or -138Q. Sixteen hours after transfection, cells were fractionated into microsomal and cytosolic fractions as previously reported. Microsomes and cytosol were further processed for immunoblotting as previously described [51].

### RT-PCR

293 cells were transfected with plasmids encoding FLAG-Nhtt17Q, -138Q, or empty vector as a negative control. Twenty-four hours after transfection, total RNA was extracted from transfected cells using TRIzol Reagent (Invitrogen) following the protocol provided by the manufacturer. RT-PCR was done as reported previously [51].

## Supporting Information

Figure S1 Mapping epitope for monoclonal anti-gp78 antibody clone 1F1 and 2G5. GST fusions of various truncations of the cytosolic tail (aa309–643) were processed for immunoblotting with monoclonal anti-gp78 antibody clone 1F2 or 2G5. Upper panel: diagramatic representation of GST fusions of gp78 mutants and summary of antibody reactions. Lower panel: 1F1 and 2G5 immunoblots.
(0.17 MB TIF)

Figure S2 gp78 does not affect ubiquitination and degradation of Nhtt588-17Q. A. HEK293 cells transfected as indicated were subjected to cycloheximide (CHX) chase. B. gp78 ubiquitinates Nhtt17Q. Nhtt17Q-expressing HEK293 cells were transfected with wt gp78, or gp78CD1/2, or empty vector as a control. Nhtt17Q was immunoprecipitated with anti-FLAG antibody. Precipitates were processed for immunoblotting for ubiquitin.
(0.18 MB TIF)
