# Supplementary figures and images for: Huntingtin Interacts with the Cue Domain of gp78 and Inhibits gp78 Binding to Ubiquitin and p97/VCP
Source: PLoS One. 2010 Jan 26;5(1):e8905. doi: 10.1371/journal.pone.0008905 (PMC2811200; doi:10.1371/journal.pone.0008905)

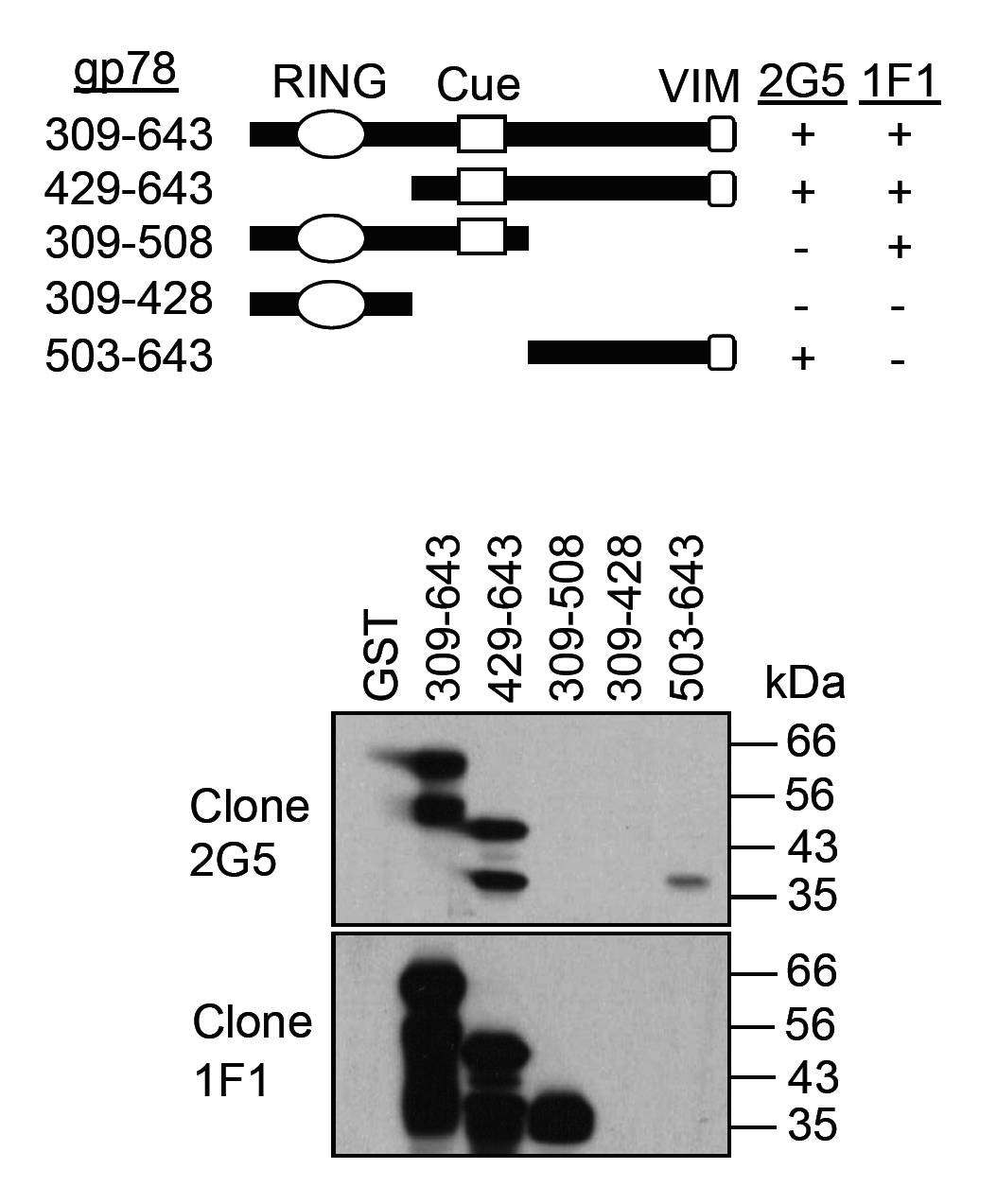

Supplement: Figure S1 — Mapping epitope for monoclonal anti-gp78 antibody clone 1F1 and 2G5. GST fusions of various truncations of the cytosolic tail (aa309–643) were processed for immunoblotting with monoclonal anti-gp78 antibody clone 1F2 or 2G5. Upper panel: diagramatic representation of GST fusions of gp78 mutants and summary of antibody reactions. Lower panel: 1F1 and 2G5 immunoblots. (0.17 MB TIF) [file pone.0008905.s001.tif]

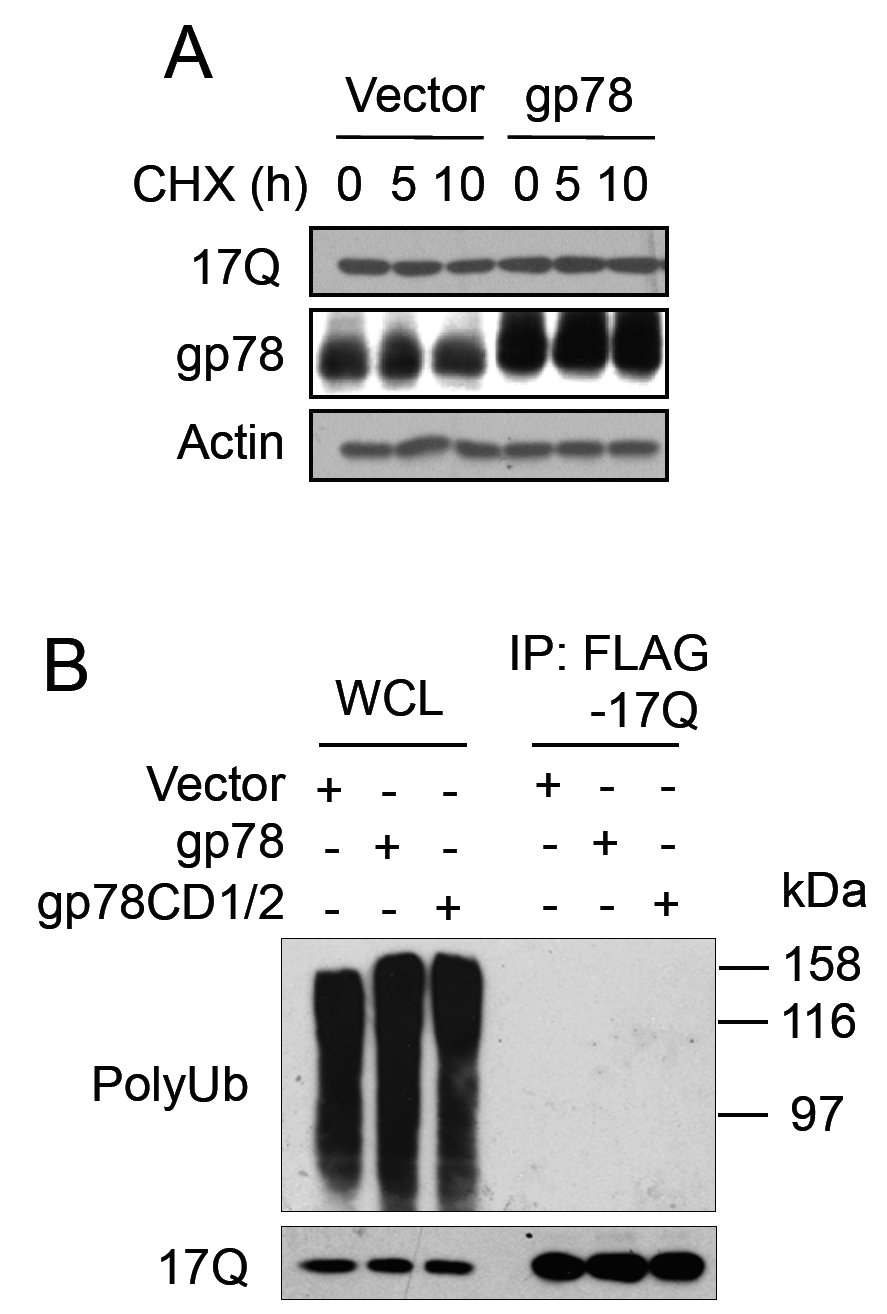

Supplement: Figure S2 — gp78 does not affect ubiquitination and degradation of Nhtt588-17Q. A. HEK293 cells transfected as indicated were subjected to cycloheximide (CHX) chase. B. gp78 ubiquitinates Nhtt17Q. Nhtt17Q-expressing HEK293 cells were transfected with wt gp78, or gp78CD1/2, or empty vector as a control. Nhtt17Q was immunoprecipitated with anti-FLAG antibody. Precipitates were processed for immunoblotting for ubiquitin. (0.18 MB TIF) [file pone.0008905.s002.tif]
